# Ovarian Cancer surgical consideration is markedly improved by the neural network powered-MIA3G multivariate index assay

**DOI:** 10.3389/fmed.2024.1374836

**Published:** 2024-05-02

**Authors:** Manjusha Roy Choudhury, Todd C. Pappas, Leo B. Twiggs, Emma Caoili, Herbert Fritsche, Ryan T. Phan

**Affiliations:** ^1^Department of Research and Development, Aspira Women’s Health, Austin, TX, United States; ^2^Division of Clinical Operations and Medical Affairs, Aspira Women's Health, Austin, TX, United States; ^3^Department of Regulatory Affairs and Quality Assurance, Aspira Women’s Health, Shelton, CT, United States; ^4^Aspira Labs, Aspira Women's Health, Austin, TX, United States

**Keywords:** ovarian cancer surgery, conservative management, pelvic mass, benign ovarian, ovarian malignancy, adnexal mass evaluation, ovarian cancer diagnosis

## Abstract

**Background:**

Surgery remains the main treatment option for an adnexal mass suspicious of ovarian cancer. The malignancy rate is, however, only 10–15% in women undergoing surgery. This results in a high number of unnecessary surgeries. A surveillance-based approach is recommended to form the basis for surgical referrals. We have previously reported the clinical performance of MIA3G, a deep neural network-based algorithm, for assessing ovarian cancer risk. In this study, we show that MIA3G markedly improves the surgical selection for women presenting with adnexal masses.

**Methods:**

MIA3G employs seven serum biomarkers, patient age, and menopausal status. Serum samples were collected from 785 women (IQR: 39–55 years) across 12 centers that presented with adnexal masses. MIA3G risk scores were calculated for all subjects in this cohort. Physicians had no access to the MIA3G risk score when deciding upon a surgical referral. The performance of MIA3G for surgery referral was compared to clinical and surgical outcomes. MIA3G was also tested in an independent cohort comprising 29 women across 14 study sites, in which the physicians had access to and utilized MIA3G prior to surgical consideration.

**Results:**

When compared to the actual number of surgeries (*n* = 207), referrals based on the MIA3G score would have reduced surgeries by 62% (*n* = 79). The reduction was higher in premenopausal patients (77%) and in patients ≤55 years old (70%). In addition, a 431% improvement in malignancy prediction would have been observed if physicians had utilized MIA3G scores for surgery selection. The accuracy of MIA3G referral was 90.00% (CI 87.89–92.11), while only 9.18% accuracy was observed when the MIA3G score was not used. These results were corroborated in an independent multi-site study of 29 patients in which the physicians utilized MIA3G in surgical consideration. The surgery reduction was 87% in this cohort. Moreover, the accuracy and concordance of MIA3G in this independent cohort were each 96.55%.

**Conclusion:**

These findings demonstrate that MIA3G markedly augments the physician’s decisions for surgical intervention and improves malignancy prediction in women presenting with adnexal masses. MIA3G utilization as a clinical diagnostic tool might help reduce unnecessary surgeries.

## 1 Introduction

Ovarian cancer is the deadliest malignancy of the female reproductive system due to late diagnosis and limited effective treatment options (1). Globally, approximately 239,000 new cases (3.6% of all cancer cases) are diagnosed, and 152,000 deaths annually (4.3% of all cancer deaths) are attributed to ovarian cancer (2). Lack of consistent symptoms during the early stages of cancer and the difficulty in differentiating cancer from benign adnexal masses contribute to poorer outcomes and lower survival rates of ovarian cancer. It has been reported that approximately 75% of ovarian cancer cases are not discovered until they have progressed to an advanced stage (3). Conversely, early detection is reported to improve patient outcomes in ovarian cancer, with a 90% 5-year survival rate compared to only 30% for those diagnosed at advanced stages (4, 5).

Pelvic masses are a common condition, occurring in approximately 5–10% of women who present with pelvic pain or other gynecologic symptoms (6, 7). Without effective diagnosis, physicians balance the risks and benefits of surgery vs. the risks of delaying treatment for potential malignancy. Due to the high risk of mortality associated with ovarian cancer, adnexal masses are typically referred to surgery for removal as the main treatment option (8, 9). Surgery is, however, both costly and comes with the risk of additional side effects, including potential negative health consequences of surgical menopause (10–12). In addition, in masses that were initially assessed as benign, the rate of spontaneous resolution was approximately 20%, whereas the combined risk of torsion, malignant transformation, and rupture was <2% (13). These data suggest that a surveillance-based approach should be considered for surgical referral in the management of an adnexal mass.

Despite the widespread use of imaging modalities such as ultrasound, computed tomography (CT) scans, and magnetic resonance imaging (MRI) for cancer detection, these imaging tools have limitations in differentiating between benign and malignant tumors (14, 15). While ultrasound is often the first-line imaging modality for adnexal masses, it is associated with a high rate of false positives and false negatives. Transvaginal ultrasound has a sensitivity ranging from 71 to 90% and a specificity ranging from 92 to 99% in the differentiation of benign and malignant ovarian masses (14–16). CT scans and MRIs have higher sensitivity and specificity for detecting adnexal masses, but they are often reserved for cases where ultrasound findings are inconclusive or when there is a high suspicion of malignancy (6, 17). A few protein biomarkers have been tested to stratify the adnexal masses, but poor performance owing to inconsistency limits their use as individual tumor markers. Serum glycoprotein biomarkers cancer antigen 125 (CA125) and human epididymis protein 4 (HE4) are commonly used in the diagnosis of ovarian cancer, as they are both generally elevated in ovarian cancer cells (18). The sensitivity and specificity of CA125 in the detection of ovarian cancer, however, are limited, with a reported sensitivity ranging from 50 to 90% and a specificity ranging from 70 to 95% (19, 20). HE4 alone has shown a sensitivity ranging from 55 to 92% and a specificity ranging from 76 to 97% (21, 22). Many studies have shown that the combination of CA125 and HE4 biomarkers improves cancer prediction (23–27).

We recently demonstrated the analytical and clinical performance of MIA3G, a deep neural network-based algorithm that incorporates the combination of CA125 and HE4 in addition to five other biomarkers, along with age and menopausal status, as input features to assess ovarian cancer risk. It was shown to have a sensitivity of 89.8% and a specificity of 84.02%, with a positive predictive value of 22.45% and a negative predictive value of 99.38% (28). In this report, we show that MIA3G markedly augments the physicians’ decision of surgical selection for women presenting with an adnexal mass and improves the malignancy prediction. These findings demonstrate that MIA3G is an important diagnostic tool supporting physicians in the clinical management of adnexal masses.

## 2 Materials and methods

### 2.1 Ethics and study datasets

All data and samples were obtained from enrolled participants for the clinical study (14–2023) and in the independent prospective study, (RP 04–2019, RP 05–2019, RP 08–2020) (29), which were carried out according to the Institutional Review Board-reviewed protocols.

Patient data and biomarkers were obtained from a multi-centered clinical study that comprised 785 enrolled patients from 12 centers. Data and sample collection protocols were identical for these studies. *Symptomatic* patients with masses were presented with pelvic pain, bloating or frequent urination, and signs of potential malignancy on imaging, for example: complex cysts, solid masses, or ascites. *Asymptomatic* patients were either discovered to have an adnexal mass on pelvic exam/imaging or those without an adnexal mass but with known genetic risk or family history of ovarian cancer.

All patients received an initial blood draw at enrollment. The study protocol directed follow-up visits consistent with the standard of care, with blood draws during the visits. Blood was drawn and batch-tested asynchronously for individual biomarkers, and the MIA3G test score was calculated (28, 29). The physicians had no access to MIA3G results in this study cohort. Physicians might have access to CA125 and/or HE4 values as part of their current clinical workup. For the data analysis reported in this manuscript, only the MIA3G value most adjacent and prior to the surgery was used for patients who underwent surgery, whereas MIA3G values from the latest blood draw were used for patients who did not have surgery.

For the independent prospective cohort, data were collected from 29 patients across 14 different centers. In this cohort, the physicians were provided with patients’ MIA3G scores to be utilized in clinical management decisions. The pathology, including tumor type if malignancy was found, and ultrasound observations were collected and analyzed along with MIA3G for the surgical cases. The participating physicians were also requested to provide satisfaction scores for the MIA3G testing outcome. The satisfaction score forms the basis of MIA3G’s impact on physicians’ decision-making process in the clinical management of patients presenting with adnexal masses.

### 2.2 Serum biomarker measurements

The serum biomarkers were measured at a CLIA-certified, CAP-accredited Aspira laboratory (Austin, TX). Briefly, a preoperative blood sample of approximately 8.5 mL was collected, and the serum was separated and collected by centrifugation within 1–6 h of blood collection. The serum sample was stored and shipped to the laboratory at 2–8°C within 8 days of collection. All serum biomarker concentrations were determined on the Roche Cobas 6,000 clinical chemistry analyzer, utilizing the c501 and e601 modules and Roche Diagnostics’ clinical assays. All measurements were performed on coded samples (blinded to patient demographics and/or pathology outcome).

### 2.3 MIA3G algorithm

MIA3G is a proprietary deep feed-forward neural network-based algorithm, and the development has been described previously (28, 29). MIA3G utilizes the clinical features of age, menopausal status, and seven biomarkers in the algorithm, which are CA125, HE4, beta-2 microglobulin, apolipoprotein A-1, transferrin, prealbumin, and follicle-stimulating hormone, to calculate the score to assess malignancy potential of adnexal masses. MIA3G outcomes are indicated as *low probability of malignancy (LP)* or *indeterminate (IND)* based on whether the MIA3G score is below or above the validated threshold, respectively (29).

### 2.4 Statistics and data analysis

Statistical analysis was performed using the R Statistical Programming Language (ver 4.2.1) (30). Python version 3 was used for all the analyses, as detailed in this paper. Python libraries NumPy and Pandas were used for analysis, the Matplotlib library was used for the bar plots, and the Seaborn library was used for the confusion matrix (31). “Percentage Malignancy Prediction Value” (% MPV) (Formula 1) is defined as the percentage probability value that a mass with a positive test is histologically malignant. For the cohort in which physicians had no access to MIA3G, % MPV was calculated as the percentage of histologically malignant cases out of the total number of patients that underwent surgery. For the independent cohort in which physicians had access to the MIA3G score, % MPV was calculated as the percentage of histologically malignant cases relative to all patients with the MIA3G *IND* score. The “accuracy” is defined as the correct predictions made by MIA3G in relation to the total number of predictions made (Formula 2) (32). The confidence interval (CI) for accuracy was recalculated using Wilson’s method (33). The “concordance” is defined as the number of correct *LP* predictions made by MIA3G in relation to the total non-malignant population (Formula 3). The CI for concordance was calculated using “proportion_confint” from the stats models’ library for the calculation of the CI of a binomial proportion (34). “Percentage Surgeries” is defined as the number of patients that underwent surgeries out of the total number of patients (Formula 4).


(1) $$\begin{array}{l} \%MalignancyPredictionValue=\\ \frac{Histologicallymalignantpatients}{\begin{array}{c} Patientswith\;MIA3G\;IND\;testresult\\ orTotalnumberofsurgeries \end{array}}\;X\;100\; \end{array}$$

(2) $$\%Accuracy=\frac{Numberofcorrectpredicitons}{Totalnumberofpredictions\;}\;X\;100$$

(3) $$\begin{array}{l} \%Concordance=\\ \frac{Numberofbenignpatientswith\;MIA3G\;LP\;testresult}{Totalnumberofnon-malignantpatients}\;X\;100 \end{array}$$

(4) $$\begin{array}{l} \%Surgeries=\\ \frac{Numberofpatientsthatunderwentsurgery}{Totalnumberofpatients}\;X\;100 \end{array}$$

## 3 Results

### 3.1 Surgery referrals reduced if physicians had access to MIA3G

**Figure 1:**
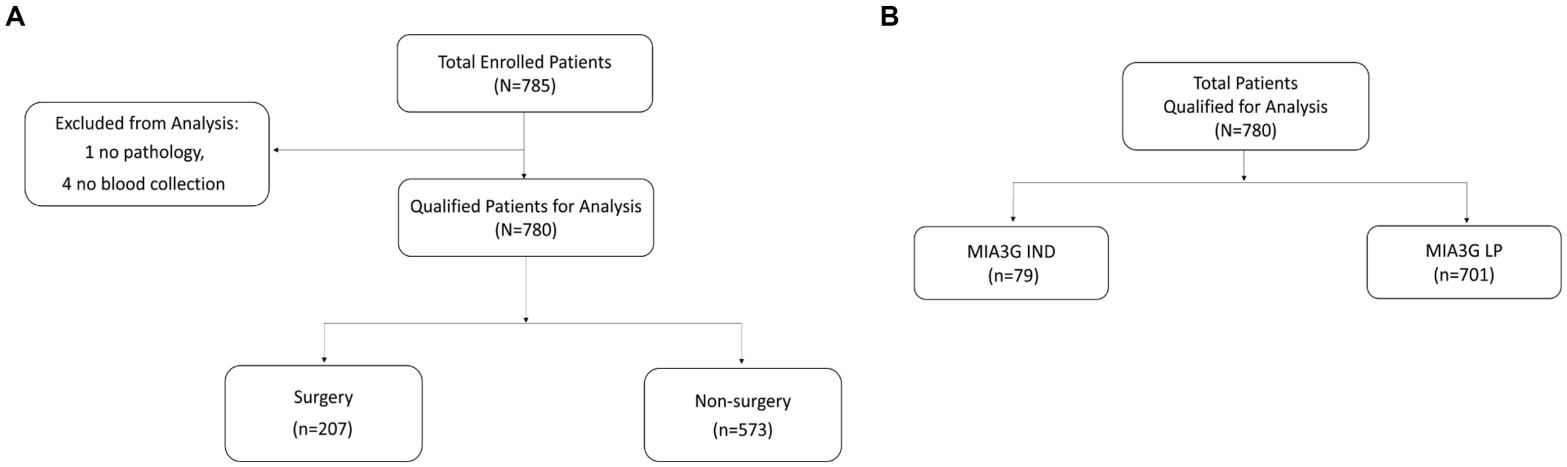
Stratification of patient samples for **(A)** actual surgery and non-surgery patients and **(B)** MIA3G risk stratification of patients.

This multi-center study comprised 785 enrolled patients from 12 centers. Table 1 shows the complete patients’ demographic and clinical data. Excluded from the analysis were four patients who had no blood draw before surgery and one patient whose post-surgery pathology information was missing. Of the 780 qualified patients, 26.5% (207) were selected for surgery by the physicians, and 73.5% (573) patients did not have surgery (Figure 1A). The physicians selected patients for surgery according to the current standard of care, including physical examination and other clinical pathological parameters, family history, imaging study, and common biomarkers (CA125 or HE4). The physicians did not have access to the MIA3G score.

**Table 1:** Patient demographics and clinical characteristics of 785 enrolled patients.

Patient characteristics	All (*N* = 785)		Benign (*n* = 191)		Malignant (*n* = 20)		No surgery (*n* = 574)	
Age	47.4 ± 12.8		49.6 ± 13.4		51.8 ± 13.0		46.5 ± 12.6	
Menopausal status	*n*	%^1^	*n*	%	*n*	%	*N*	%
Pre-/Peri-	508	64.7	105	55.0	8	40.0	395	68.8
Post-	277	35.3	86	45.0	12	60.0	179	31.2
Race/ethnicity	*n*	%	*n*	%	*n*	%	*N*	%
White	515	65.6	142	74.3	13	65.0	360	62.7
African American	74	9.4	19	9.9	1	5.0	54	9.4
Asian	36	4.6	14	7.3	5	25.0	17	3.0
Ashkenazi Jewish	8	1.0	2	1.0	1	5.0	5	0.9
Native American/Hawaiian	5	0.6	0	0.0	0	0.0	5	0.9
Other/mixed	6	0.8	3	1.6	0	0.0	3	0.5
Unknown	141	18.0	11	5.8	0	0.0	130	22.6
Clinical symptoms	n	%	n	%	n	%	N	%
Asymptomatic	328	41.8	68	35.602	7	35.0	251	43.7
Symptomatic	457	58.2	121	63.351	13	65.0	323	56.3
Clinical classification			n	%				
Endometriosis			22	11.52				
Mature teratoma			18	9.42				
Mucinous tumor			18	9.42				
Serous tumor			30	15.71				
Unknown/other			103	53.93				
Ovarian cancer stage					n	%		
Stage 1					7	35.0		
Stage 2					2	11.8		
Stage 3					1	5.9		
NS					7	41.2		
Non-ovarian surgery	N	%	N	%	N	%		
	11	1.4	8	4.2	3	15		

^1^Percentages are calculated as a portion of the stratified group (all, benign, malignant, and no-surgery).

**Figure 2:**
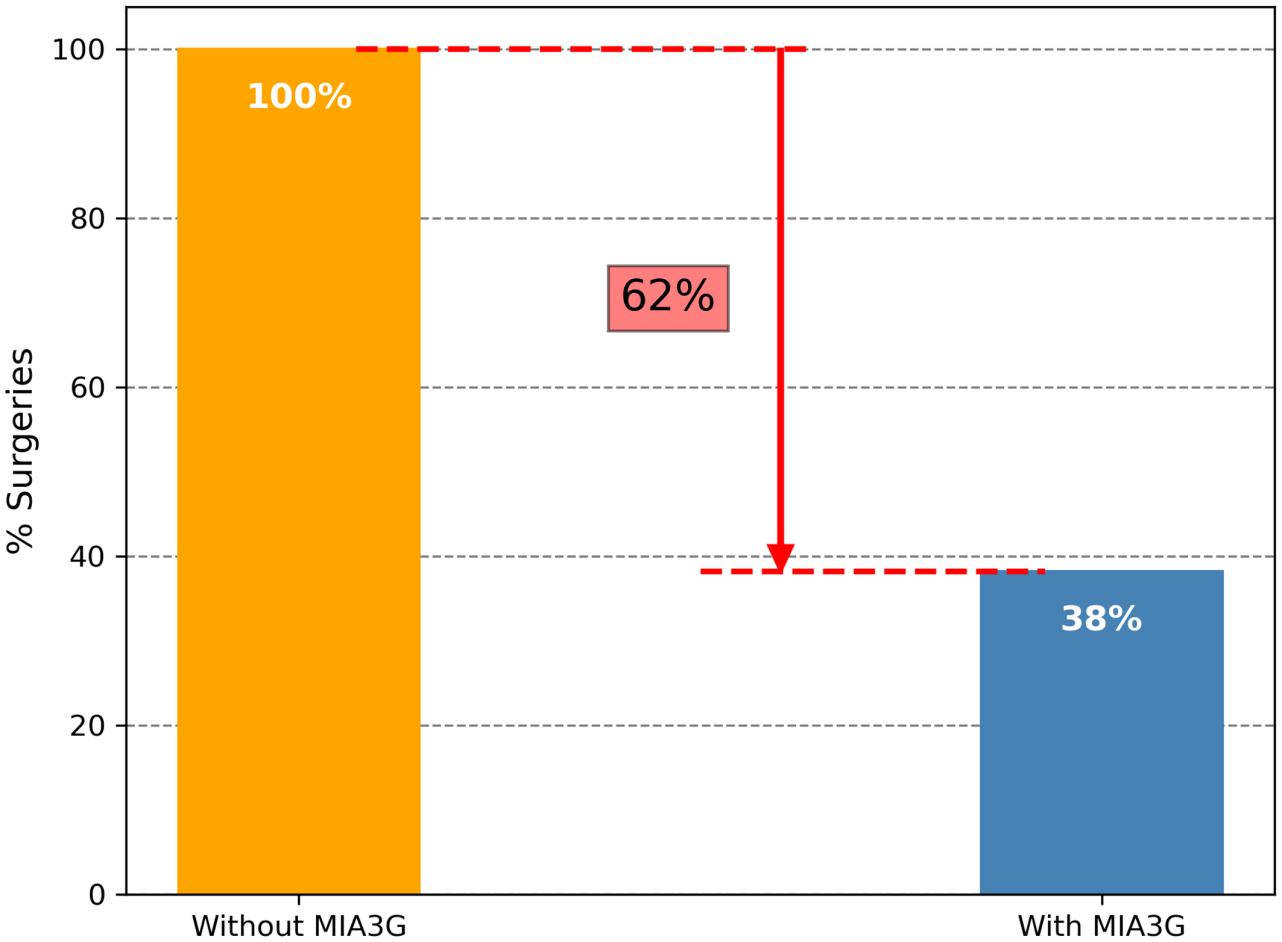
Differential surgery referrals without and with MIA3G stratification. The orange bar depicts surgery referrals without MIA3G (considered as 100%), and the blue bar depicts surgery referrals with MIA3G stratification (normalized to 100%).

We calculated the MIA3G score for each qualified patient as previously described (28, 29). The validated MIA3G stratified the malignancy risk of a suspicious adnexal mass, based on an “at risk” score from 0 to 10, into the LP (MIA3G <5.0) or IND (MIA3G ≥ 5.0) category, with negative predictive value >99% (28). While LP-stratified patients continued to be monitored, the IND-stratified patients were recommended for additional workup, including surgical consideration. In this context, the MIA3G analysis indicated that 89.9% (701) patients were in the LP category, whereas only 10.1% (79) patients were in the IND category (Figure 1B). Assuming that all IND patients would have been putative candidates for surgical management, the maximum surgical referral rate based on MIA3G values would be 10.1%, compared to 26.5% of actual surgery performed. This resulted in a reduction of surgery referrals by 61.9% if the physicians had access to the MIA3G scores (Figure 2).

To further understand this trend of potential surgery referral reduction in different patient cohorts, we calculated the MIA3G score and determined the total IND patients for each subcategory according to the symptomatic presentation, menopausal status, and age (Table 2). The analysis revealed that had the physicians had access to MIA3G value, the surgery referrals would have been reduced by 63% among the symptomatic patients (48/453 MIA3G IND cases vs. 131/453 actual surgery cases) and 59% among the asymptomatic patients (31/327 MIA3G IND cases based on MIA3G vs. 76/327 actual surgery cases). Similar analysis showed that surgery referral would have been reduced by 77% in premenopausal patients vs. 45% of postmenopausal patients and reduced by 70% in patients < 55 years old vs. 46% of ≥ 55-year-old patients if the physicians had access to MIA3G scores (Table 2).

**Table 2:** Differential surgery reduction by the MIA3G stratification of individual patient cohorts.

	Total	Surgery	Expected surgery MIA3G IND	Differential percentage reduction in surgery by MIA3G
Total	780	207	79	62%
Based on symptoms				
Symptomatic	453	131	48	63%
Asymptomatic	327	76	31	59%
Based on menopausal status				
Premenopausal	505	111	26	77%
Postmenopausal	275	96	53	45%
Based on age group				
Age ≤ 55	596	139	42	70%
Age > 55	184	68	37	46%

### 3.2 Malignancy prediction value significantly increased by MIA3G

Surgery consideration for an adnexal mass suspicious of ovarian cancer is primarily because of malignancy risk. Of the 207 surgery patients (196 ovarian surgeries and 11 non-ovarian surgeries, Supplementary Table S1), 19 masses (9.2%) were found to be malignant, based on surgical pathology results (Table 3). Patients were further stratified based on symptoms, menopausal status, and age groups, and the distribution of malignant patients in the different cohorts is shown in Table 3. The MIA3G scores were calculated for these surgery patients, and the total MIA3G IND patients were calculated for each classified category (Table 3). MIA3G values indicated 39 IND cases (18.8%) among a total of 207 surgery patients.

**Table 3:** Malignancy prediction improvement stratified by MIA3G for individual patient cohorts.

	Surgery	MIA3G IND	Malignancies^*^	Increase in malignancy prediction value
Total	207	39	19	431%
Based on symptoms				
Symptomatic	131	26	12	404%
Asymptomatic	76	13	7	485%
Based on menopausal status				
Premenopausal	111	9	7	1,133%
Postmenopausal	96	30	12	220%
Based on age group				
Age ≤ 55	139	18	14	672%
Age > 55	68	21	5	224%

*Malignancies confirmed by surgical pathology studies.

**Figure 3:**
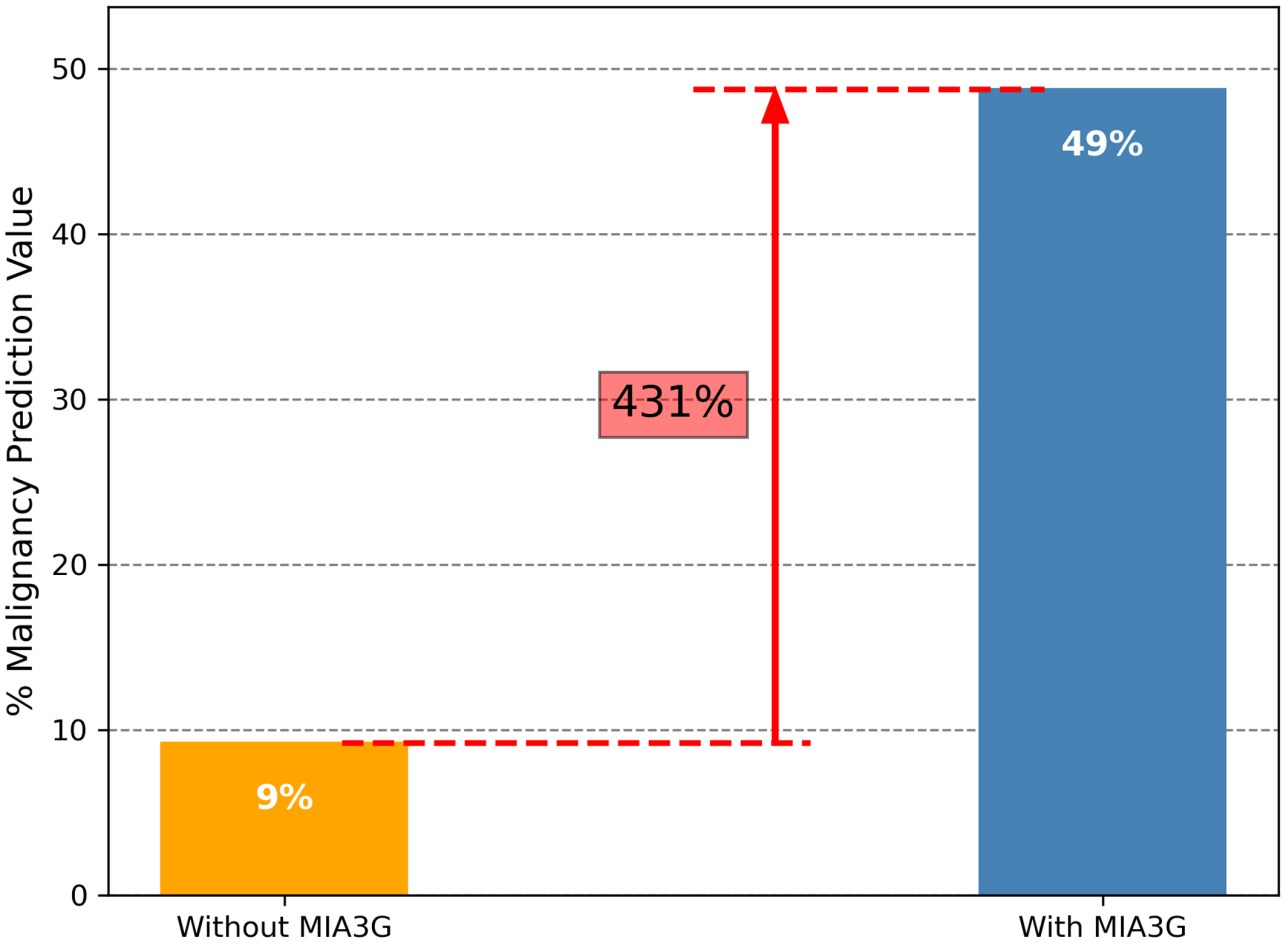
Malignancy prediction value without (orange bar) and with MIA3G (blue bar).

To determine the impact of MIA3G in supporting physician’s surgical selection, we evaluated MIA3G malignancy prediction. The MPV was determined according to Formula 1 – Statistics and Data Analysis section. The analysis showed that the MPV for actual surgery cases was 9.18% (19/207) vs. 48.72% (19/39) if surgery was referred by MIA3G IND stratification. This result indicates a significant improvement in malignancy prediction by 431% if the physicians were supported by MIA3G (Figure 3). When comparing MPV for actual surgery cases vs. MIA3G IND cases as demonstrated in Table 3 and partitioning by clinical characteristics, similar trends of drastic malignancy prediction improvement were observed, ranging from 220% to over 1,130% regardless of symptoms, menopausal status, or age groups, had the physicians had access to MIA3G values.

### 3.3 MIA3G improves the accuracy of surgical selection

**Figure 4:**
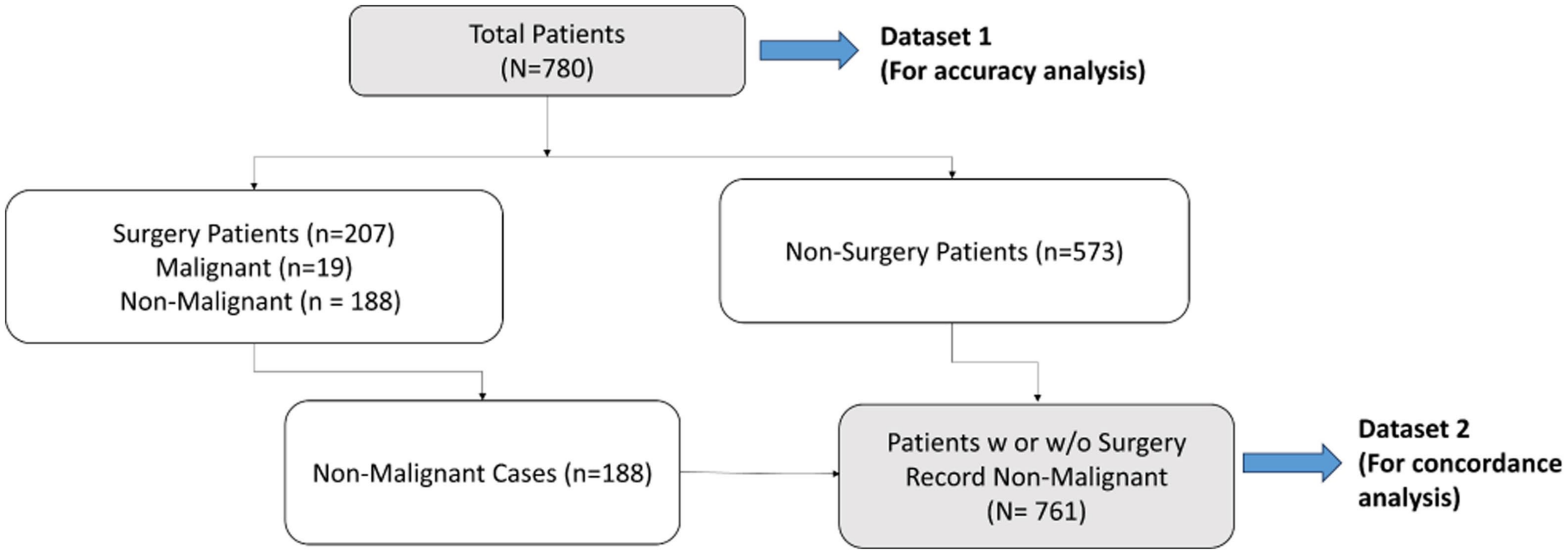
Flowchart of datasets used for accuracy and concordance studies – Dataset 1 (total population) to be used for accuracy study, and Dataset 2 (non-malignant cases) to be used for concordance study.

Figure 4 describes the workflow diagram for the two datasets from the multi-centered study. The first dataset (Dataset 1) contains all 780 patients (non-malignant and malignant), and a second dataset (Dataset 2) contains patients who were either surgically found to be non-malignant or presumed non-malignant since no surgery was referred and all had been observed for more than a 7-month period (35) from the first date of observation or blood draw (*N* = 761).

**Figure 5:**
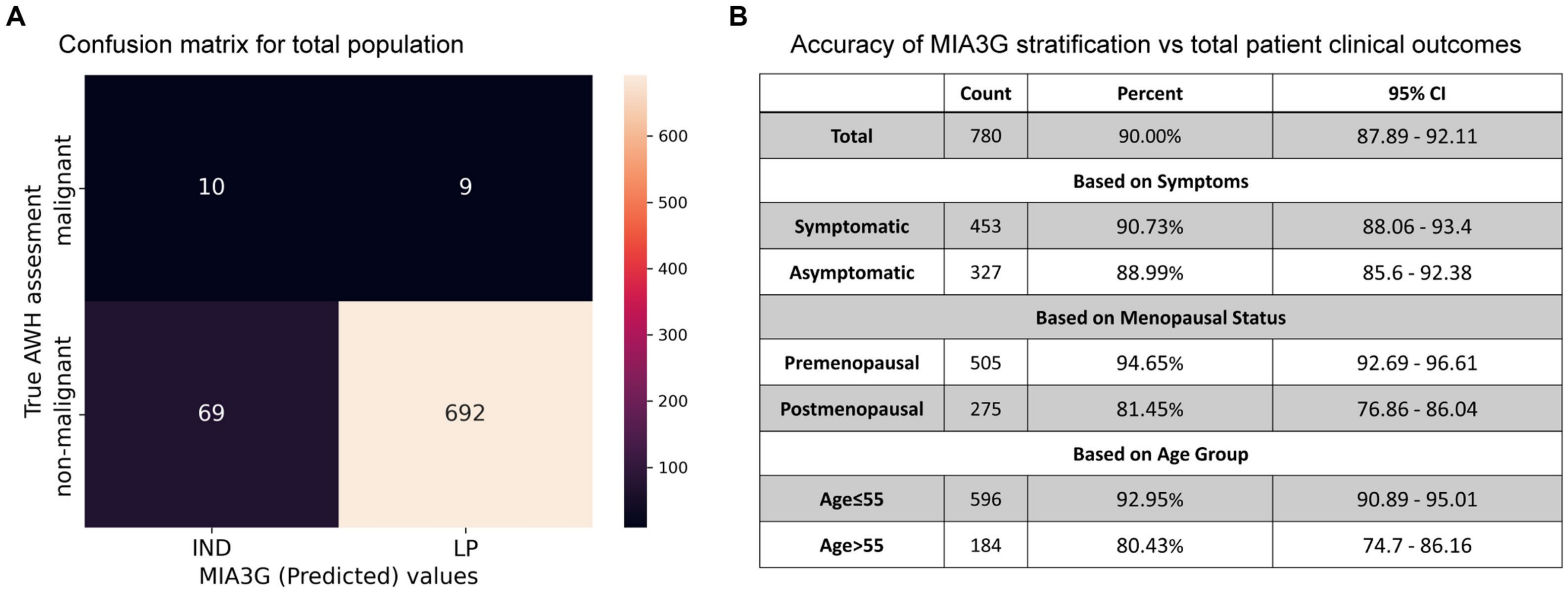
**(A)** Confusion matrix generated for the total population with clinical outcome vs. MIA3G stratification. **(B)** Accuracy of MIA3G for each subcategorized patient cohort.

To determine the accuracy, we examined true positive (malignancy) and true negative (non-malignancy) among the total analyzed patients (Dataset 1). The accuracy of surgical selection was calculated as indicated by Formula 2 – Statistics and Data Analysis. Of the 780 analyzed patients, 207 went to surgery, and 19 malignant cases were found. This resulted in 75.9% accuracy of surgery referral by physicians (19 accurate malignancies +573 non-surgery patients out of a total of 780 patients). Had physicians had access to MIA3G, as seen in the confusion matrix in Figure 5A, MIA3G would correctly label 10 malignant and 692 non-malignant patients out of a total of 780 patients. The accuracy of MIA3G for the surgery referral was 90% (95% CI of 87.89–92.11) (Figure 5B). Of note, further explanations of the nine malignant patients that were incorrectly classified as LP by MIA3G are provided in Supplementary Table S2.

The MIA3G accuracies for surgery referrals were further determined based on symptoms, menopausal status, and age. Additional information is available in Supplementary Figure S1. The high accuracy was found to be similar among symptomatic patients (90.73%) (CI 88.06–93.4%) and asymptomatic patients (88.99%) (CI 85.6–92.38) (Figure 5B). MIA3G showed higher accuracy in the premenopausal cohort at 94.65% (CI 92.69–96.61) compared to the postmenopausal cohort at 81.45% (CI 76.86–86.04). Similarly, MIA3G showed higher accuracy in patients ≤55 years of age (92.95%; CI 90.89–95.01) compared to patients aged>55 years (80.43%; CI 74.7–86.16) (Figure 5B). These analyses indicate that MIA3G improves the accuracy of surgery referrals if physicians had access to MIA3G scores.

### 3.4 Concordance studies on non-malignant cohorts

**Figure 6:**
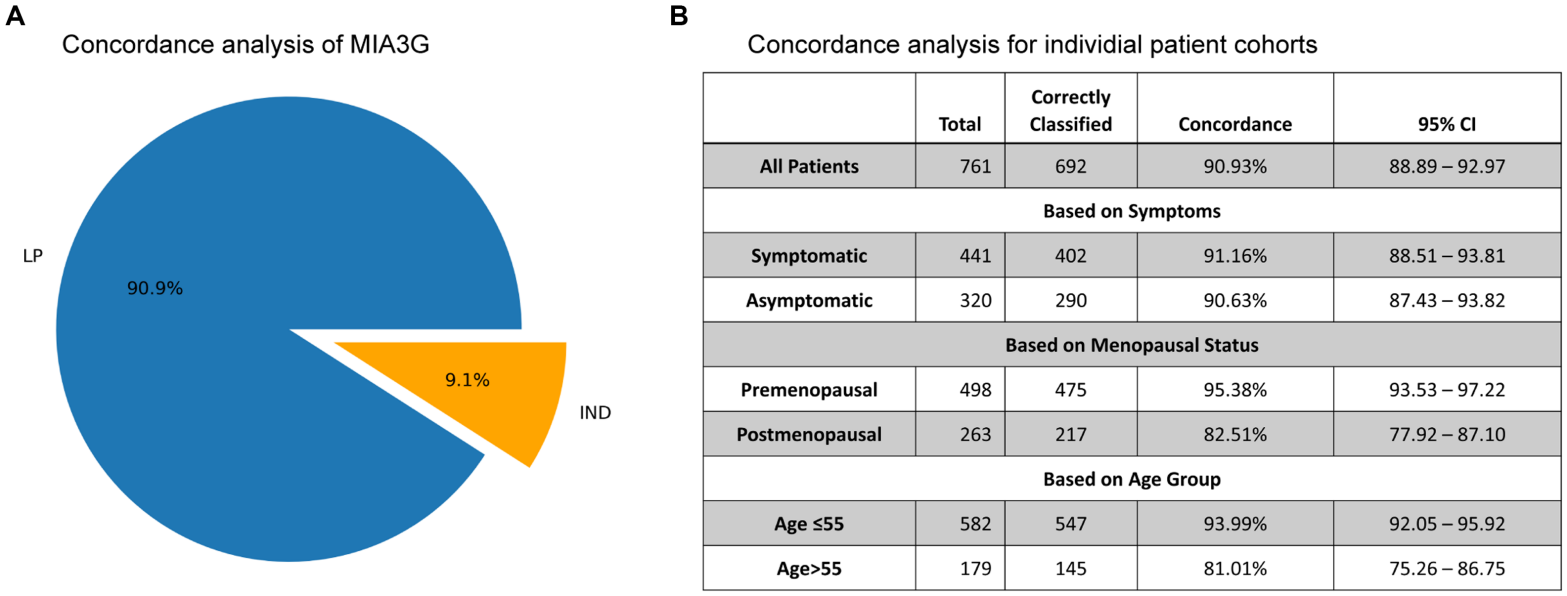
**(A)** The pie chart depicts the concordance of MIA3G stratification for non-malignant cases. **(B)** Concordance analysis of MIA3G for each subcategorized patient cohort.

Concordance analysis is described by Formula 3 (see Statistics and Data Analysis section) for all 761 non-malignant cases in Dataset 2, comprising 188 surgically confirmed non-malignant and 573 presumed non-malignant due to no surgery (Figure 4). For actual surgical selection, concordance was found to be 75.3% (573 non-surgery patients out of 761 non-malignant cases). As presented in Figure 6A, concordance for MIA3G-based surgical referral was 90.9%, in which MIA3G stratified 692 (90.9%) patients as LP out of the 761 non-malignant cases. Of the 69 non-malignant patients classified as IND, 39 (56.5%) were symptomatic and 29 (42%) had surgical procedures.

The concordance values were further determined based on symptoms, menopausal status, and age (Figure 6B). The results mirrored the trends observed in the accuracy of MIA3G in surgical selection. High MIA3G concordance was observed in the symptomatic patients at 91.16% (CI 88.51–93.81%) and in the asymptomatic patients at 90.63% (CI 87.43–93.82%). MIA3G concordance was greater in the premenopausal cohort (95.38%; CI 93.53–97.22) compared to the postmenopausal cohort (82.51%; CI 77.92–87.10). Similarly, MIA3G concordance was greater in patients ≤55 years old [93.99% (CI 92.05–95.92)] compared to patients with age > 55 years [81.01% (CI 75.26–86.75)]. These data indicate that MIA3G also improves the concordance for non-malignancy consideration if physicians had access to MIA3G scores.

### 3.5 Validation of MIA3G’s role in surgical referrals

An independent multicenter real-world clinical practice study was initiated in the first 3 months of implementing MIA3G in clinics across the US. In this study, physicians ordered the MIA3G assay as part of the clinical workup and utilized MIA3G scores to support surgery selection for their patients. Patient’s surgical pathology correlation was obtained if surgery was selected (Supplementary Table S3). A physicians’ satisfaction survey was also requested to evaluate MIA3G’s impact on patient care.

Of a total of 357 patients from more than 70 centers, 29 patients from 14 centers with complete clinical data were available at the time of data analysis (Table 4). Of these 357 evaluable patients, 87% (311) were stratified as MIA3G LP and 13% (46) as IND. These results were comparable with previously reported malignancy rates observed in patients undergoing ovarian cancer surgery (36, 37). The composition of the patients distributed in the independent study is summarized in Table 4.

**Table 4:** Patient demographics and clinical characteristics in the prospective independent cohort.

Patient characteristics	All (*N* = 29)		Benign (*n* = 1)		Malignant (*n* = 0)		No surgery (*n* = 28)	
Age	47.0 ± 16.7		53.0 ± 0.0		0.0 ± 0.0		46.8 ± 16.9	
Menopausal Status	*n*	%^1^	*n*	%	*n*	%	*n*	%
Pre-/Peri-	15	51.7	0	0	0	0	15	53.6
Post-	14	48.3	1	100	0	0	13	46.6
Race/Ethnicity	*n*	%	*n*	%	*n*	%	*n*	%
White	16	55.2	0	0	0	0	16	57.1
African American	1	3.4	0	0	0	0	1	3.6
Asian	1	3.4	0	0	0	0	1	3.6
Hispanic	1	3.4	0	0	0	0	1	3.6
Sephardic Jewish	1	3.4	0	0	0	0	1	3.6
Unknown	9	31	1	100	0	0	8	28.6
MIA3G score	*n*	%	*n*	%	*N*	%	*n*	%
LP	28	96.6	1	100	0	0	27	96.4
IND	1	3.4	0	0	0	0	1	3.6

^1^Percentages are calculated as a portion of the stratified group (all, benign, malignant, and no-surgery).

**Figure 7:**
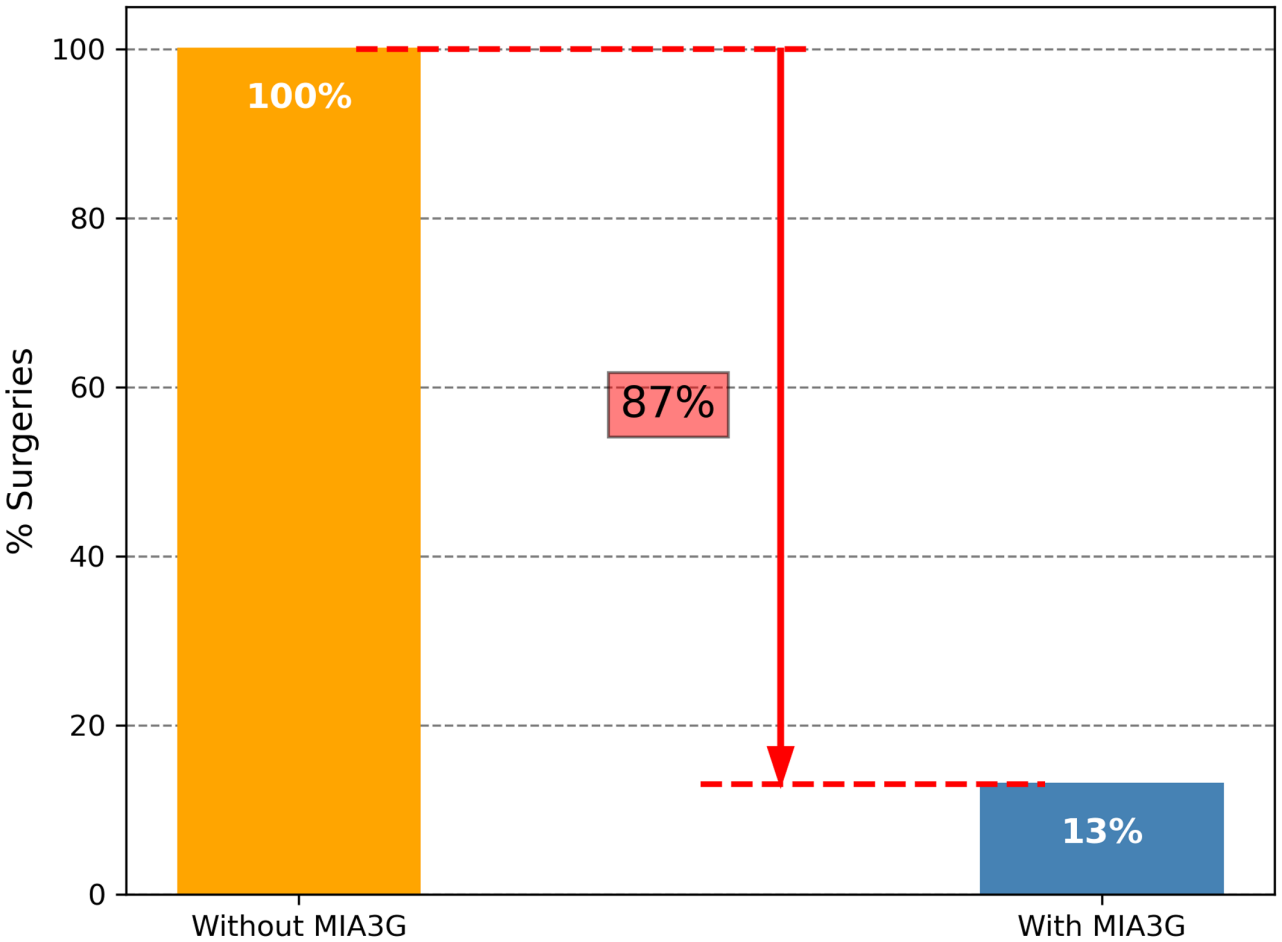
Comparison of surgery referrals when physicians had no access to MIA3G (orange bar) vs. physicians utilizing MIA3G stratification (blue bar) in real-world clinical practice.

Of the 29 analyzed cases, 1 patient (Accession A0104511, Supplementary Table S3) was selected for surgery by the clinician. The MIA3G score, however, stratified this case as LP, and the surgical pathology study found the mass pathologically benign. The MIA3G score stratified one patient (Accession A0106586) as IND. The physician, however, decided not to select this patient (A0106586) for surgery, pending additional clinical follow-up. Altogether, the surgery consideration for this cohort was only 3.4% (1/29) and equivalent to the 3.4% (1/29) patients being stratified as IND by MIA3G, although the statistical significance cannot be determined due to the small sample size. Compared to the large study cohort of 780 patients, in which 207 surgeries were selected with no MIA3G participation, there was an 87% decrease in surgery procedures when physicians had access to MIA3G in real-world clinical practice (Figure 7).

Satisfaction surveys were collected from 19 physicians across 14 sites (serving 29 patients) (Supplementary Table S4). The results indicated that 6 (31.6%) physicians were highly satisfied and 11 (57.9%) physicians were satisfied with MIA3G’s testing outcomes. Of the 19 physicians, 7 (36.8%) were highly satisfied, 6 (31.5%) were satisfied, and 4 (21.1%) were neutral about MIA3G’s impact on clinical management. These initial reviews suggest that physicians have a favorable impression of MIA3G utilization in clinical practice.

## 4 Discussion

MIA3G is a neural network-powered multivariate index analysis tool designed to assess the malignancy risk of a presumed non-malignant or IND adnexal mass. This study demonstrated that MIA3G may show utility in informing surgical referrals where malignancy risk is suspected in adnexal mass patients. The study found a critical predictive reduction in surgeries for symptomatic (63%), premenopausal (77%), and younger women (70%) with pathologically benign masses. MIA3G scores in conjunction with physician assessment were shown to significantly improve malignancy predictive values by over 400%, which were also notably improved in relevant stratified cohorts. Retrospective studies indicated MIA3G improves the accuracy of physician’s clinical management for an adnexal mass from 75% to over 90%. Concordance between pathology and clinical outcomes and MIA3G for a benign subset population showed that 90.9% of patients were correctly classified as LP. Furthermore, surgeries were reduced by 87% in patients from our real-world study cohort, where physicians had access to MIA3G prior to surgery referrals during initial clinical assessment and management. Taken together, these findings were notable as they show that MIA3G as a diagnostic tool may help inform surgical considerations for ovary removal and/or diagnostic surgeries.

We chose to investigate the impact of MIA3G on surgical selection in this large study cohort after the patient’s surgery had been decided to ensure that physicians utilized current standard clinical practice without being influenced by MIA3G. To this end, the finding that a physician’s decision for surgery selection markedly improved by MIA3G was clinically important. Over 60% of surgeries would be reduced if the physicians had access to MIA3G. Surgery reduction was found irrespective of the patient’s clinical characteristics, and importantly, even if all MIA3G-stratified IND cases were presumed to be putative candidates for surgical consideration. While LP-stratified patients continue to be monitored, IND-stratified patients are recommended for additional workups, in which surgical consideration is one of the potential options. In this context, had the physicians appropriately utilized the MIA3G recommendation, not all MIA3G IND patients would have been selected for surgery and the rate of surgery reduction would be even higher. In fact, this hypothesis is validated in our real-world study cohort, in which surgeries were reduced by 87% when physicians had access to MIA3G prior to surgery referrals.

To further illustrate the impact of MIA3G in supporting physicians’ surgical selection, we evaluated the MIA3G MPV. The study found that the current standard clinical practice has an MPV of 9.18% and significantly increases to 48.72% if surgery referrals are considered by MIA3G IND stratification (Figure 3). We previously reported the analytical and clinical performance of MIA3G stratifying patients based on malignancy risk with a negative predictive value of over 99% (28, 29). In this study, the concordance for MIA3G in surgical referrals was over 90% for all non-malignant cases. These significant findings confirm that MIA3G performance markedly improves the clinical management of adnexal mass.

Although surgery remains the main option for the management of adnexal masses, a recent large-scale ovarian cancer screening trial found that over 60% of the masses in women resolved on subsequent visits and serial observation (38). Moorman et al. found that up to 36% of women who underwent adnexal surgery did not have ovarian cancer (39). In fact, ovarian cancer is rare, with a global diagnosis of approximately 240,000 new cases, with rates varying by country (2). Surgery is costly and comes with the risk of additional side effects, including potential negative health consequences of surgical menopause (10–12). The high volume of adnexal surgeries can also lead to surgical backlogs and increased patient waiting times. In this regard, avoiding needless diagnostic surgical procedures can help improve patient quality of life, thus, highlighting the need for better preoperative risk assessment and non-invasive diagnostic tools such as MIA3G to support physicians in making surgical referrals for patients presenting with adnexal masses.

It is important to note that our study presumed that surgical referrals by physicians for the analyzed patients were primarily due to malignancy risk. In addition to the primary concern of malignancy, surgical removal of adnexal masses is occasionally considered to relieve uncomfortable symptoms for patients. Moreover, patients who did not undergo surgery in this study were considered benign since all had been observed for more than a 7-month period (35). In addition, we also postulated that all MIA3G IND patients were candidates for surgery referral, although surgical consideration was only one of the recommended options for the IND-stratified patients (28, 29). Although these assumptions were made to simplify the analysis, there was no evidence that the assumptions statistically affected the study findings and conclusions. In fact, the findings were validated in the real-world cohort in which physicians had access to MIA3G scores prior to surgery referrals (Figure 7).

Finally, it should be noted that our real-world cohort in this study was limited to 357 patients, of whom only 29 had complete information for analysis at the time this manuscript was written. This cohort is part of a larger, more focused study to evaluate the utility of MIA3G in the clinical management of adnexal masses. For future studies, it would also be pertinent to include a thorough data collection that records the type of management surgery performed (oophorectomy or ovarian cystectomy), the physician’s reasons/influences for management choice, and the histology of the benign mass, if applicable. Incorporating these stringent inclusion criteria may better address those assumptions in the current study.

In a personalized approach utilizing the patient’s specific biomarkers, imaging studies, and other clinical characteristics, MIA3G serves as an effective clinical tool to support physicians in assessing the risk of ovarian cancer and considering appropriate management options for women with adnexal masses. MIA3G augments a physician’s decision for surgery referral and helps reduce unnecessary ovary removal surgeries.

## Data availability statement

The original contributions presented in the study are included in the article/Supplementary material, further inquiries can be directed to the corresponding authors.

## Ethics statement

The studies involving humans were approved by Institutional Review Board Services, ADVARRA Inc. The studies were conducted in accordance with the local legislation and institutional requirements. The participants provided their written informed consent to participate in this study.

## Author contributions

MR: Conceptualization, Data curation, Formal analysis, Investigation, Methodology, Software, Validation, Writing – original draft, Writing – review & editing. TP: Conceptualization, Data curation, Formal analysis, Investigation, Methodology, Validation, Writing – original draft, Writing – review & editing. LT: Data curation, Investigation, Validation, Visualization, Writing – review & editing. EC: Data curation, Investigation, Validation, Visualization, Writing – review & editing, Project administration. HF: Formal analysis, Investigation, Methodology, Validation, Writing – review & editing. RP: Conceptualization, Data curation, Formal analysis, Funding acquisition, Investigation, Methodology, Project administration, Resources, Software, Supervision, Validation, Visualization, Writing – original draft, Writing – review & editing.
