# Supplementary material for: Ovarian Cancer surgical consideration is markedly improved by the neural network powered-MIA3G multivariate index assay
Source: Front Med (Lausanne). 2024 May 2;11:1374836. doi: 10.3389/fmed.2024.1374836 (PMC11097110; doi:10.3389/fmed.2024.1374836)
Supplement: Supplementary file 1 [file Table_2.DOCX]

Supplementary Material

**Supplementary Table 1.** MIA3G prediction and pathology details for non-ovarian surgeries.

| Participant ID | Age | Menopausal Status | Symptomatic | Surgery | MIA3G Score | MIA3G Risk | Pathology Finding | Benign/  Malignant |
| --- | --- | --- | --- | --- | --- | --- | --- | --- |
| N009 | 64 | postmenopausal | asymptomatic | yes | 0.6 | LP | Leiomyoma | Benign |
| OVA1429733 | 48 | premenopausal | symptomatic | yes | 4.4 | LP | p53 signature lesion & walthard nest in fallopian tube = no evidence of serous tubal intraepithelial carcinoma **unknown** | Benign |
| OVA1446524 | 47 | premenopausal | symptomatic | yes | 0.4 | LP | Adenomyosis Leiomyoma | Benign |
| OVA1488700 | 43 | premenopausal | symptomatic | yes | 0.4 | LP | Adenomyosis Leiomyoma | Benign |
| OVN1000110 | 42 | premenopausal | symptomatic | yes | 0.5 | LP | Leiomyosarcoma | Malignant |
| OVN1000374 | 43 | premenopausal | symptomatic | yes | 1.5 | LP | Fibrin & mixed inflammatory cells | Benign |
| OVN1000439 | 62 | postmenopausal | symptomatic | yes | 9.8 | IND | Stage IV uterine papillary serous carcinoma (AJCC 8th Ed: T1aN0M1) Side note: neoadjuvant chemo (carboplatin & taxol) - 3 cycles | Malignant |
| OVN1000445 | 34 | premenopausal | symptomatic | yes | 0.4 | LP | Leiomyoma | Benign |
| N079 | 51 | premenopausal | symptomatic | yes | 0.6 | LP | Leiomyoma | Benign |
| N110 | 52 | premenopausal | asymptomatic | yes | 3.0 | LP | Endometriod adenocarcinoma FIGO Gr1 (AJCC 8th Ed: T1aN0) Myometrium w/ adenomyosis & leiomyomata Side note: patient diagnosed with Lynch Syndrome | Malignant |
| OVN1000326 | 23 | premenopausal | symptomatic | yes | 0.7 | LP | Masses "spontaneously resolved" | Benign |

**Supplementary Table 2.** Pathological characterization of malignant cases misclassified by MIA3G as low probability of malignancy (LP; n=9).

| Participant ID | Age | Menopausal  Status | Symptomatic | Surgery | MIA3G  Score | Histology | Stage | Comments |
| --- | --- | --- | --- | --- | --- | --- | --- | --- |
| N048 | 19 | premenopausal | symptomatic | yes | 0.4 | Sertoli-Leydig  cell | IC2 | less than 0.5% of all ovarian cancers |
| OVN1000062 | 44 | premenopausal | symptomatic | yes | 1.7 | Epithelial: endometrioid | I | Early stage |
| N068 | 55 | postmenopausal | asymptomatic | yes | 0.6 | Benign: Mintue Leydig  cell tumor & fallopian tube paratubal cyst |  | Rare histopathology type |
| N075 | 52 | postmenopausal | asymptomatic | yes | 0.4 | Epithelial:  Serous | II | Early stage |
| OVA1472180 | 80 | postmenopausal | asymptomatic | yes | 2.9 | Epithelial  carcinosarcoma | IA | Non epithelial |
| OVA1479784 | 54 | postmenopausal | asymptomatic | yes | 1.8 | Nonepithelial  Granulosa cell | I | not common less than 10% of cancers |
| N039 | 72 | postmenopausal | asymptomatic | yes | 4.0 | Serous Carcinoma involving left fallopian tube & left ovary with surface involvement (STIC) – AJCC 8^th^ Ed. T1c2N0 | I, Gr. 3 | Early stage |
| OVN1000110 | 42 | premenopausal | symptomatic | yes | 0.5 | Leiomyosarcoma |  | Sarcoma |
| N110 | 52 | premenopausal | asymptomatic | yes | 3.0 | Endometrioid adenocarcinoma (uterus) | FIGO Gr. 1 | Endometrial Cancer |

**Supplementary Table 3.** MIA3G stratification and clinical and surgery outcomes of 29 patients in the prospective independent study.

| Accession | MIA3G  Score | MIA3G  Risk | Was surgery performed?  (Yes/No) | Pathology if  surgery was performed?  (Benign/Malignant/N/A) | If the mass was malignant, what was the tumor type?  (or N/A) | Did the findings correlate with ultrasound observations? (Yes/No/N/A) |
| --- | --- | --- | --- | --- | --- | --- |
| A0106361 | 3.4 | LP | No | N/A | N/A | N/A |
| A0106362 | 1.7 | LP | No | N/A | N/A | N/A |
| A0106363 | 0.4 | LP | No | N/A | N/A | N/A |
| A0107072 | 0.6 | LP | No | N/A | N/A | N/A |
| A0107695 | 1.1 | LP | No | N/A | N/A | N/A |
| A0109421 | 0.4 | LP | No | N/A | N/A | N/A |
| A0108843 | 0.4 | LP | No | N/A | N/A | N/A |
| A0108357 | 0.5 | LP | No | N/A | N/A | N/A |
| A0106966 | 0.6 | LP | No | N/A | N/A | N/A |
| A0108143 | 0.5 | LP | No |  |  |  |
| A0108532 | 3.1 | LP | No |  |  |  |
| A0102431 | 0.7 | LP | No |  |  |  |
| A0103028 | 0.5 | LP | No |  |  |  |
| A0107816 | 0.4 | LP | No |  |  |  |
| A0108621 | 0.4 | LP | No |  |  |  |
| A0108360 | 0.4 | LP | No | N/A | N/A | Yes |
| A0109518 | 1.8 | LP | No | N/A | N/A | N/A |
| A0107823 | 2.0 | LP | No | N/A | N/A | Yes |
| A0103482 | 3.8 | LP | No | N/A | N/A | Yes |
| A0108233 | 0.4 | LP | No | N/A | N/A | Yes |
| A0108019 | 0.4 | LP | No | N/A | N/A | Yes |
| A0105609 | 0.9 | LP | No | N/A | N/A | Yes |
| A0108240 | 0.6 | LP | No | N/A | N/A | Yes |
| A0106586 | 7.8 | I | No | N/A | N/A | Yes |
| A0104511 | 0.5 | LP | Yes | Benign | N/A | Yes |
| A0104092 | 1.0 | LP | No |  |  |  |
| A0108144 | 0.4 | LP | No |  |  |  |
| A0106872 | 0.4 | LP | No |  |  |  |
| A0102839 | 0.4 | LP | No |  |  |  |

**Supplementary Table 4.** Physicians' satisfaction survey outcomes.

| **SATISFACTION**  **OUTCOMES** | **TOTAL PHYSICIANS (N=19; 14 SITES)** | | | | |
| --- | --- | --- | --- | --- | --- |
|  | **Very Satisfied** | **Satisfied** | **Neutral** | **Needs**  **Improvement** | **No Answer** |
| **Experience with MIA3G** | **6** | **11** |  |  | **2** |
| **MIA3G impact on patient management** | **7** | **6** | **4** |  | **2** |


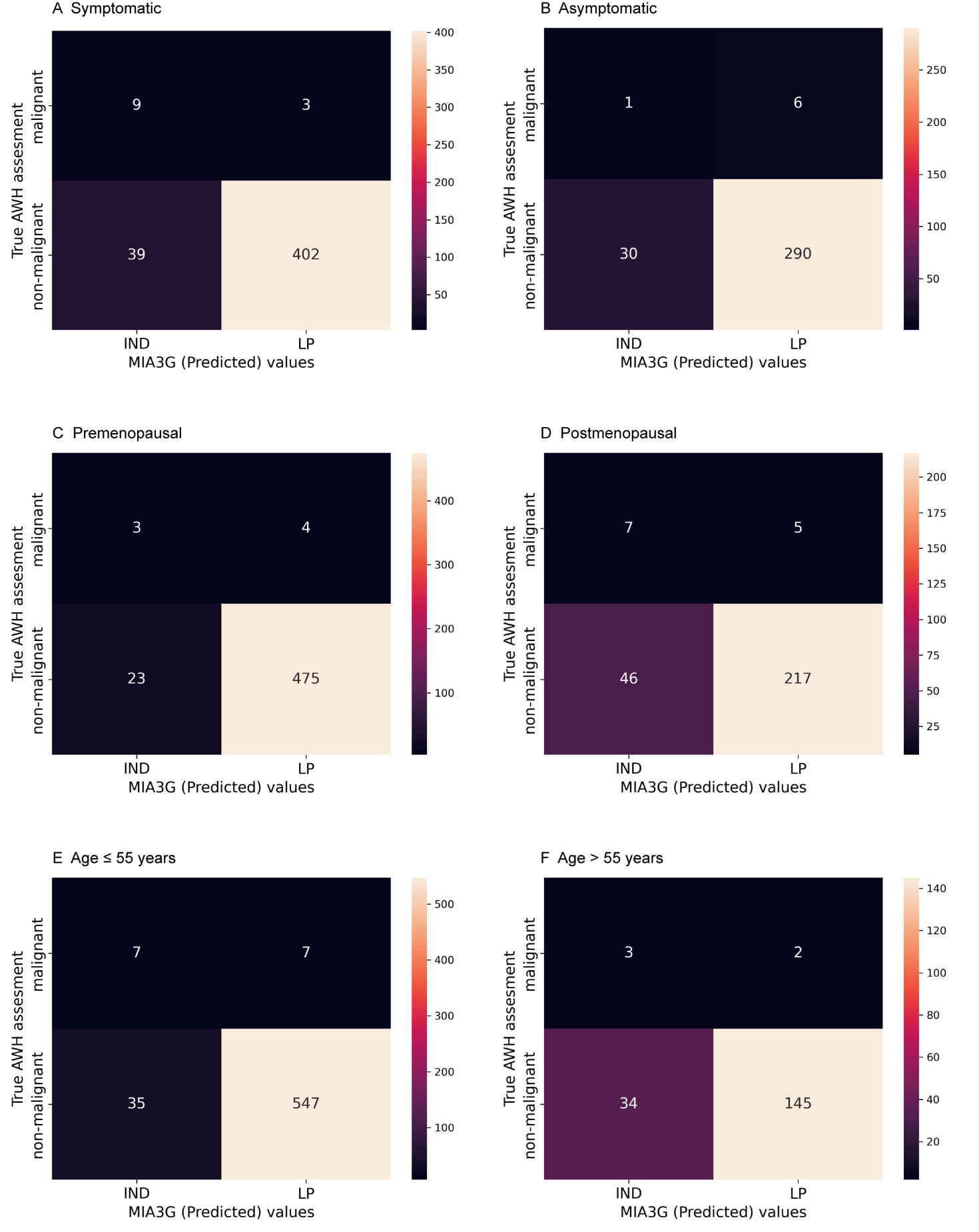


**Supplementary Figure 1.** Confusion matrix for individual cohorts in the total population (N=780) for **(A)** Symptomatic Patients (n=453), **(B)** Asymptomatic Patients (n=327), **(C)** Premenopausal (n=505), **(D)** Postmenopausal (n=275), **(E)** Age ≤ 55 years (n=596), and **(F)** Age > 55 years (n=184).
